# Trehalose-6-phosphate synthase regulates chitin synthesis in *Mythimna separata*

**DOI:** 10.3389/fphys.2023.1109661

**Published:** 2023-02-13

**Authors:** Hong-Jia Yang, Meng-Yao Cui, Xiao-Hui Zhao, Chun-Yu Zhang, Yu-Shuo Hu, Dong Fan

**Affiliations:** College of Plant Protection, Northeast Agricultural University, Harbin, China

**Keywords:** *Mythimna separata*, trehalose-6-phosphate synthase, chitin, trehalose, RNAi

## Abstract

Trehalose is a substrate for the chitin synthesis pathway in insects. Thus, it directly affects chitin synthesis and metabolism. Trehalose-6-phosphate synthase (TPS) is a crucial enzyme in the trehalose synthesis pathway in insects, but its functions in *Mythimna separata* remain unclear. In this study, a *TPS*-encoding sequence in *M. separata* (*MsTPS*) was cloned and characterized. Its expression patterns at different developmental stages and in diverse tissues were investigated. The results indicated that *MsTPS* was expressed at all analyzed developmental stages, with peak expression levels in the pupal stage. Moreover, *MsTPS* was expressed in the foregut, midgut, hindgut, fat body, salivary gland, Malpighian tubules, and integument, with the highest expression levels in the fat body. The inhibition of *MsTPS* expression *via* RNA interference (RNAi) resulted in significant decreases in the trehalose content and TPS activity. It also resulted in significant changes in *Chitin synthase* (*MsCHSA* and *MsCHSB*) expression, and significantly decrease the chitin content in the midgut and integument of *M. separata*. Additionally, the silencing of *MsTPS* was associated with a significant decrease in *M. separata* weight, larval feed intake, and ability to utilize food. It also induced abnormal phenotypic changes and increased the *M. separata* mortality and malformation rates. Hence, *MsTPS* is important for *M. separata* chitin synthesis. The results of this study also suggest RNAi technology may be useful for enhancing the methods used to control *M. separata* infestations.

## 1 Introduction

Trehalose, which is an important blood sugar in insects, accounts for 80%–90% of the carbohydrate content in the insect hemolymph. It is present in almost all insect tissues and is a crucial source of energy (Hottiger et al., 1987). Trehalose synthesis is an important process affecting insect growth and development, physiological homeostasis, ovum formation, and energy metabolism (Santos et al., 2012). In insects, trehalose is synthesized mainly through the TPS/TPP pathway. Specifically, trehalose-6-phosphate is a product of a reaction between uridine diphosphate glucose and glucose-6-phosphate catalyzed by trehalose-6-phosphate synthase (TPS). Trehalose-6-phosphate phosphatase (TPP) then converts trehalose-6-phosphate to trehalose (Giaever et al., 1988; Strom and Kaasen, 1993; Shukla et al., 2015; Yang et al., 2017). Previous studies revealed that trehalose is synthesized in the fat body of insects (Shukla et al., 2015; Yang et al., 2017). Subsequent research confirmed that *TPS* genes are highly expressed in insect fat body (Chen et al., 2020). TPS is a key enzyme in the trehalose synthesis pathway in insects. Although TPP has been detected in some insects, most insects contain only TPS (Tang et al., 2014). To date, the *TPS* sequences in more than 50 insect species have been cloned (Tang et al., 2018). Among insects, *Drosophila melanogaster* was the first to have its *TPS* gene cloned (Chen et al., 2002), after which the *TPS* genes of *Locusta migratoria* (Cui and Xia, 2009), *Leptinotarsa decemlineata* (Shi et al., 2016), *Diaphorina citri* (Song et al., 2021) and other insects were cloned. These genes encode proteins comprising approximately 800 amino acids, with two highly conserved TPS and TPP domains.

The synthesis, transformation, and modification of chitin are critical for insect growth and development. Trehalose is considered to be the main substrate for chitin synthesis (Shi et al., 2016; Xiong et al., 2016). The silencing of *TPS* genes in insects can affect the trehalose and chitin contents, while also leading to abnormal molting, growth, and development, and may even lead to death. In an earlier study, after *TcTPS* expression was inhibited in *Tribolium castaneum*, the *CHS1* expression level increased significantly at 72 h, whereas the *CHS2* expression level decreased significantly at 48 and 72 h. Moreover, some of the tested insects were unable to pupate normally and their chitin content decreased (Chen et al., 2018). Another study demonstrated that the inhibition of *SeTPS* expression in *Spodoptera exigua* leads to a decrease in the trehalose content as well as mortality rates of 50.94% and 66.76% at 48 and 204 h, respectively (Tang et al., 2010). The injection of *Bactrocera minax* larvae with dsRNA to silence *BmTPS* expression reportedly significantly decreases the TPS activity, trehalose content, and the expression of three key genes in the chitin biosynthesis pathway, ultimately resulting in an abnormal phenotype and a mortality rate of 52% (Xiong et al., 2016).

*Mythimna separata* (Lepidoptera: Noctuidae) is a polyphagous insect that mainly infests corn, wheat, rice, sorghum, and other food crops. It is distributed worldwide (Zhang et al., 2018). At sufficiently high densities, *M. separata* can seriously decrease agricultural production. In this study, we identified a *TPS* cDNA sequence in the *M. separata* transcriptome database. The spatiotemporal expression pattern of *MsTPS* was analyzed. Furthermore, *MsTPS* was functionally characterized on the basis of RNA interference (RNAi) experiments. The results of this study may be useful for developing new methods for controlling *M. separata*.

## 2 Materials and methods

### 2.1 Insect rearing

*Mythimna separata* larvae were collected from Xiangyang Station of Northeast Agricultural University (Harbin, China) and transferred to an artificial climate chamber for the rearing of several generations. The incubation conditions were as follows: temperature, 25°C ± 1°C; humidity, 60%–70%; photoperiod, 14-h light:10-h dark cycle. Larvae were fed fresh corn leaves and adults were fed 5% honey water until they laid eggs.

### 2.2 Cloning of *MsTPS* cDNA

Various larval instars were collected and flash-frozen in liquid nitrogen for the subsequent transcriptome sequencing, which was performed by Annoroad Gene Technology company (Beijing, China). The *M. separata* transcriptome database was screened for the *TPS* sequence, which was then analyzed and compared with the sequences in the NCBI database.

Total RNA was extracted from fourth instar larvae using TRIzol (Invitrogen, Carlsbad, CA, United States) according to the manufacturer’s instructions. First-strand cDNA was synthesized from 1.0 µg total RNA using the ReverTra Ace qPCR RT Master Mix with gDNA Remover (Toyobo, Shanghai, China). On the basis of the screened sequence, primers *MsTPS*-F and *MsTPS*-R (Supplementary Table S1) were designed using the Primer Premier 5.0 software for the PCR amplification of the *MsTPS* sequence. The synthesized cDNA served as the template for the PCR. The PCR amplification product was then purified using the Monarch Gel Extraction kit (NEB, Ipswich, MA, United States), inserted into the Trans1-T1 cloning vector (TransGen Biotech, Beijing, China), and sequenced to confirm its accuracy.

### 2.3 Amino acid sequence analysis and phylogenetic tree construction

The obtained *MsTPS* sequence was registered and deposited in the NCBI database (https://www.ncbi.nlm.nih.gov/). The *MsTPS* open reading frame was predicted and translated using ORF Finder (https://www.ncbi.nlm.nih.gov/orffinder/). The isoelectric point and molecular weight of the encoded amino acid sequence were predicted using the Expasy Compute pI/Mw online tool (https://www.expasy.org/). Its conserved domains were detected using the SMART online program (http://smart.embl-heidelberg.de/). Additionally, 31 known insect TPS amino acid sequences were downloaded from the NCBI database for the construction of a phylogenetic tree according to the maximum likelihood method with best-fitting model of MEGA7.0, with 1,000 bootstrap replicates (Kumar et al., 2016).

### 2.4 Spatiotemporal *MsTPS* expression pattern

Total RNA was extracted from insects at different developmental stages (i.e., first-day first to sixth instar larvae, prepupae, pupae, and adults) and from various tissues (i.e., foregut, midgut, hindgut, fat body, salivary gland, Malpighian tubules, and integument). The *MsTPS* expression levels in different *M. separata* developmental stages and tissues were analyzed by quantitative real-time PCR (qRT-PCR), with *Msβ-actin* (GenBank accession number: GQ856238) and *MsGAPDH* (glyceraldehyde-3-phosphate dehydrogenase; GenBank accession number: HM055756) used as reference genes. Primer Premier 5.0 was used to design qRT-PCR primer pairs *MsTPS*-q-F and *MsTPS*-q-R, *Msβ-actin*-q-F and *Msβ-actin*-q-R, and *MsGAPDH*-q-F and *MsGAPDH*-q-R (Supplementary Table S1). The qRT-PCR mixture comprised 2 µl cDNA, 6.8 µl ddH_2_O, 0.6 µl sense and anti-sense primers, and 10 µl THUNDERBIRD SYBR qPCR Mix kit (Toyobo, Shanghai, China). The qRT-PCR was performed using the SimpliAmp PCR instrument (Thermo Fisher Scientific, MA, United States of America), with the following PCR conditions: 95°C for 3 min; 40 cycles of 94°C for 10 s and 57°C for 30 s. The data were recorded using the Bio-Rad CFX Manager 3.1 software. Melting curves were checked to assess the specificity of the qRT-PCR. Moreover, primer efficiency was validated before analyzing gene expression. The qRT-PCR was completed using three technical replicates and three biological replicates.

### 2.5 SiRNA synthesis and interference

For the RNAi analysis, siRNA sequences for *MsTPS* (GCG​UUA​CAG​GAA​CAG​GUU​UTT and AAA​CCU​GUU​CCU​GUA​ACG​CTT) as well as for the negative control (UUC​UCC​GAA​CGU​GUC​ACG​UTT and ACG​UGA​CAC​GUU​CGG​AGA​ATT) were synthesized (GenePharma, Shanghai, China) (Guo et al., 2018). The siRNA sequences were diluted and dissolved in DEPC-treated water for a final concentration of 20 µM. The first-day fourth instar larvae were injected with 2 µl siRNA for *MsTPS* or the negative control using a microsyringe. The larvae were fed normally after the injection.

### 2.6 Post-RNAi *MsTPS* expression

Larvae were collected at 3, 6, 12, 24, 48, 72, and 96 h after the injection of siRNA. Total RNA was extracted from the larvae and then reverse transcribed to cDNA for an analysis of *MsTPS* expression by qRT-PCR.

### 2.7 Effect of *MsTPS* silencing on *chitin synthase* gene expression

To explore the effect of *MsTPS* silencing on the expression of *MsCHSA* (GenBank accession number: KT948989) and *MsCHSB* (GenBank accession number: KY348776), Primer Premier 5.0 was used to design qRT-PCR primer pairs *MsCHSA*-q-F and *MsCHSA*-q-R as well as *MsCHSB*-q-F and *MsCHSB*-q-R (Supplementary Table S1). The *MsCHSA* and *MsCHSB* expression levels were analyzed using the cDNA that was used for the post-RNAi examination of *MsTPS* expression.

### 2.8 Post-RNAi TPS activity

Larvae collected at 3, 6, 12, 24, 48, 72, and 96 h after the injection of siRNA were used to measure the MsTPS activity according to the instructions for the Insect TPS Enzyme-linked Immunoassay (ELISA) kit (Jiangsu Meibiao Biotechnology Co., Ltd., Jiangsu, China). The protein concentrations of the samples were determined on the basis of Coomassie brilliant blue staining as previously described (Bradford, 1976), with a standard curve prepared using bovine serum albumin (Takara, Dalian, China). Briefly, 20 µl each sample and 180 µL Coomassie brilliant blue dye (Takara) were added to a 96-well plate, which was then incubated at room temperature for 5 min before the absorbance at 595 nm was measured.

### 2.9 Post-RNAi trehalose content

A previously reported method was used to determine the trehalose content (Steele, 1988; Ge et al., 2011) of three replicates of larvae collected at 3, 6, 12, 24, 48, 72, and 96 h after the injection of siRNA. A standard curve was prepared with trehalose concentrations of 0.05, 0.1, 0.2, 0.4, 0.6, 0.8, and 1.6 mmol/L. Each sample was placed in a sterilized homogenizer and then ground after 1 mL PBS (pH 7.4) was added. The samples were centrifuged at 5,000 *g* for 20 min at 4°C. The supernatants were collected and added to a 96-well plate. Following the addition of 5 µl 1% H_2_SO_4_ solution to each well, the 96-well plate was heated at 90°C for 10 min and then cooled on ice. Next, 5 µl 30% KOH solution was added to each well and then the 96-well plate was heated at 90°C for 10 min. After cooling the 96-well plate on ice, 100 µl chromogenic agent (0.02 g anthranone in 10 ml 80% H_2_SO_4_ solution) was added to each well, after which the 96-well plate was heated at 90°C for 10 min and then inserted into a microplate reader (Tecan, Hombrechtikon, Switzerland). The absorbance at 630 nm was recorded for each sample. The protein concentrations of the samples were determined as previously described (Bradford, 1976).

### 2.10 Post-RNAi chitin contents in the midgut and integument

A previously reported method was used to determine the chitin content (Arakane et al., 2005) of larvae collected and weighed at 3, 6, 12, 24, 48, 72, and 96 h after the injection of siRNA. Each analysis was performed using three replicates of 15 larvae. The larvae were dissected in normal saline to obtain the midgut and integument samples, which were placed in a 1.5-mL centrifuge tube and then ground to a powder in liquid nitrogen. After adding 500 µl 6% KOH solution, the ground samples were heated at 80°C for 90 min and then centrifuged at 12,000 *g* for 20 min at 4°C. The supernatant was discarded and each sample was suspended in 1 mL PBS buffer and then centrifuged at 12,000 *g* for 20 min at 4°C. After discarding the supernatant, each sample was resuspended in 200 µl Mcllvaine buffer (pH 6.0) (Tianzd, Beijing, China). To hydrolyze chitin to *N*-acetyl glucosamine (GlcNAc), 50 µl chitinase from *Streptomyces griseus* (Sigma-Aldrich, Shanghai, China) was added to individual samples, which were then incubated for 72 h at 37°C.

The GlcNAc concentrations were determined using a modified Morgan-Elson assay (Reissig et al., 1955). Briefly, various GlcNAc concentrations (0.00025, 0.0005, 0.001, 0.002, 0.004, and 0.008 mol/L) were used as standards. The samples incubated for 72 h were centrifuged at 12,000 *g* for 20 min at 4°C. For each sample, 10 µl supernatant and different GlcNAc standards were added to new centrifuge tubes. Next, 10 µL 0.27 mol/L sodium tetraborate was added to individual samples, which were then heated at 99.9°C for 10 min. The samples were immediately cooled to room temperature and then mixed with 100 µl 10% DMAB solution (10 g p-dimethylaminobenzaldehyde in a solution comprising 12.5 ml concentrated hydrochloric acid and 87.5 ml glacial acetic acid, diluted 1:10 with glacial acetic acid). The samples were heated at 37°C for 20 min and then centrifuged at 12,000 *g* for 5 min at 4°C. An 80-µl aliquot of each sample was transferred to a 96-well plate, and the absorbance at 585 nm was recorded. Standard curves were prepared using the different GlcNAc concentrations.

### 2.11 Effects of *MsTPS* silencing on the ability of *M. separata* to utilize food

Fourth instar larvae were injected with 2 µL siRNA targeting *MsTPS* using a microsyringe. The control larvae were injected with 2 µl negative control siRNA. The larvae were subsequently fed normally (i.e., fresh corn leaves). At 3, 6, 12, 24, 48, 72, and 96 h after the injection of siRNA, larvae, remaining corn leaves, and excreta were placed in a 70°C oven and dried until they reached a constant weight. The following indices were recorded: 1) fresh weight of the larvae before the experiment; 2) fresh weight of the corn leaves before feeding; 3) dry weight of the remaining corn leaves for each time-point; 4) dry weight of the excreta (Zhao et al., 2017). The analysis was completed using three replicates of 15 larvae.

To determine the dry weight of the larvae before the experiment, 45 larvae from the same batch were selected at the beginning of the experiment and divided into three replicates. The fresh weight of the larvae was recorded. The larvae were then dried to obtain the dry weight. The larval dry weight:fresh weight ratio was calculated. The dry weight of the larvae before the experiment was calculated according to the dry weight:fresh weight ratio. The dry weight of the fresh corn leaves before feeding was similarly determined.

The ability of insects to digest and use food was evaluated on the basis of nutritional indices, including increase in dry weight W), larval feed intake B), relative growth rate (RGR), relative consumption rate (RCR), efficiency of the conversion of digested food (ECD), efficiency of the conversion of ingested food (ECI), and approximate digestibility (AD). These indices were calculated as follows (Waldbauer, 1968; Mole and Zera, 1993).

• W (g) = dry weight of larvae after the experiment − dry weight of larvae before the experiment
• B (g) = leaf dry weight before feeding − leaf dry weight after feeding
• RGR [g∙(g∙h)^−1^] = W/(dry weight of larvae before the experiment × experiment time)
• RCR [g∙(g∙h)^−1^] = B/(dry weight of larvae before the experiment × experiment time)
• ECD (%) = [W/(B− dry weight of excreta)] × 100
• ECI (%) = (W/B) × 100
• AD (%) = [(B− dry weight of excreta)/B] × 100

### 2.12 Effects of *MsTPS* silencing on *M. separata* growth and development

Fourth instar larvae were injected with 2 µl siRNA targeting *MsTPS* using a microsyringe. The control larvae were injected with 2 µl negative control siRNA. The larvae were subsequently fed normally (i.e., fresh corn leaves). Five replicates were prepared, with 30 larvae per group. The larvae were examined every 6 h. The time of death, the time of each molting, and the weight after each molting were recorded to detect abnormal molting. Finally, the mortality rates at 24, 48, and 72 h were calculated. The time required for the fifth instar larvae, sixth instar larvae, and pupae to develop was recorded. The weight of the fifth instar larvae, sixth instar larvae, and pupae was determined. The rates of abnormal molting and pupation were calculated.

The Helicon Focus digital photograph depth-of-field processing tool and the Helicon Remote camera control software were used to photograph and analyze (5–20 focal planes) the insects that exhibited abnormal development.

### 2.13 Statistical analysis

The qRT-PCR data were analyzed according to a published 2^−ΔΔCT^ method (Jo et al., 2002). Statistical analysis was carried out with GraphPad Prism 9.3.0 software. Outliers were removed using Grubbs’ test (GraphPad online software). Shapiro-Wilk’s test was used to check normality in distribution. Resulting pairs were compared using the Student’s t-test. One-way ANOVA was applied to determine the significant differences (*p* < 0.05) for different groups by using the Tukey’s test. Then the results were plotted using the software GraphPad Prism 9.3.0.

## 3 Results

### 3.1 Analysis of the *MsTPS* cDNA and amino acid sequences

A novel *M. separata TPS* cDNA sequence (*MsTPS*; GenBank accession number: MN832898) was identified on the basis of transcriptome sequencing data. A sequence analysis revealed that the *MsTPS* cDNA is 4,551 bp long, with an open reading frame comprising 2,490 bp. The encoded protein (829 amino acids) has an isoelectric point of 6.68 and a molecular weight of 93.01 kDa. Moreover, it includes two conserved domains, namely, TPS (amino acids 19–499) and TPP (amino acids 537–762).

### 3.2 Phylogenetic analysis of MsTPS

In the phylogenetic tree constructed for MsTPS and other insect TPS sequences, MsTPS was clustered with the TPS of *S. exigua* (ABM66814), *Spodoptera litura* (XP_022814358), and *Spodoptera frugiperda* (XP_035433508), indicative of a relatively close relationship. It was also clustered with the TPS of *Helicoverpa armigera* (XP_021201246), *Bombyx mori* (XP_004926812), *Manduca sexta* (XP_030020846), *Papilio xuthus* (XP_013178239), *Antheraea pernyi* (ARD05072), and *Heortia vitessoides* (AYO46920). In contrast, MsTPS was distantly related to the TPS of *Aphis glycines* (QII15889) (Supplementary Figure S1). The clustering of TPS from various species was basically consistent with the morphological classification of these insects.

### 3.3 Spatiotemporal *MsTPS* expression pattern

**FIGURE 1:**
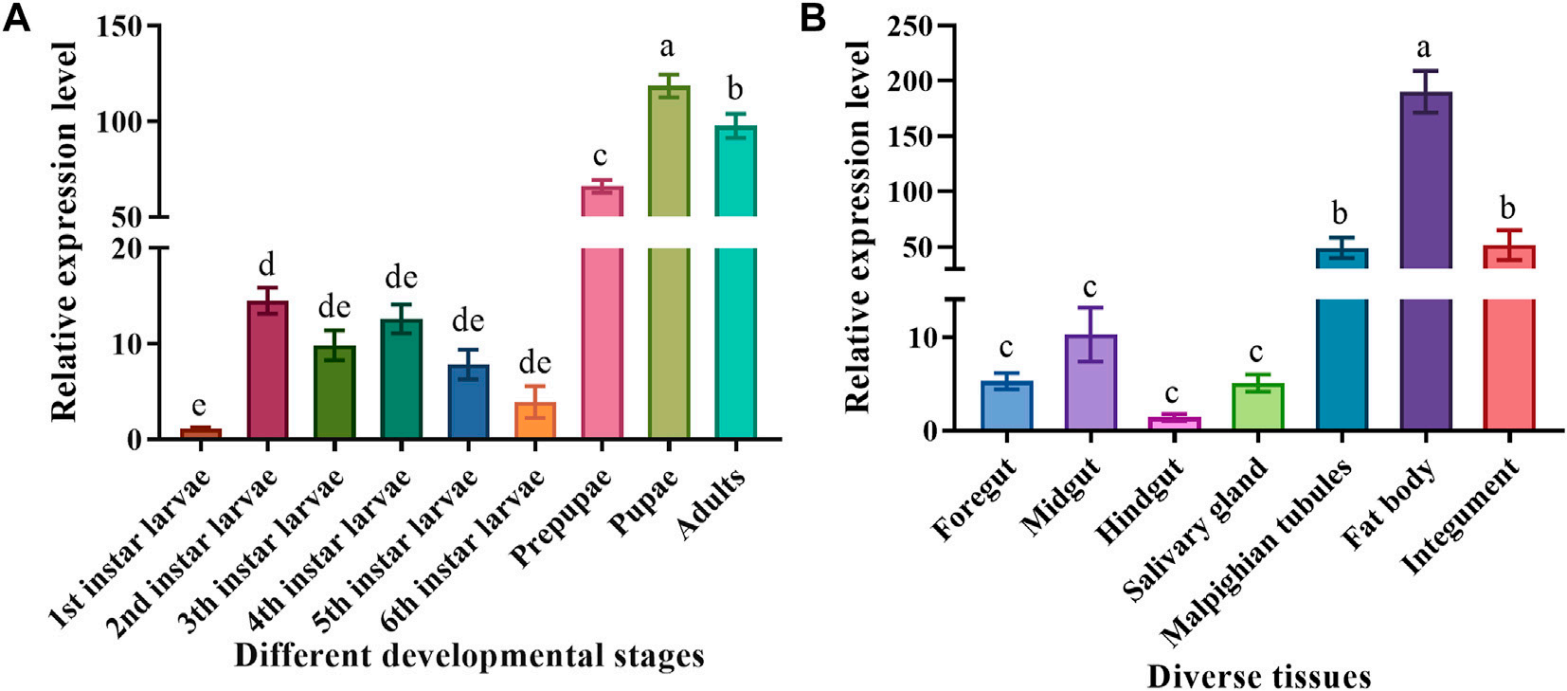
Spatiotemporal *MsTPS* expression pattern in different developmental stages **(A)** and in diverse tissues **(B)** of *M. separata*. The qRT-PCR data for *MsTPS* were analyzed using the 2^−ΔΔCT^ method to determine relative expression levels, which were calculated as the mean ± SE. The expression data were normalized on the basis of the geometric mean of the expression of two reference genes (encoding *β*-actin and glyceraldehyde-3-phosphate dehydrogenase). The GraphPad Prism software was used for the data analysis. Different letters indicate significant differences (*p* < 0.05) according to the Tukey’s test.

To clarify the *MsTPS* expression pattern during different *M. separata* developmental stages, the larvae, prepupae, pupae, and adults were analyzed by qRT-PCR. The results showed that *MsTPS* was expressed in all examined *M. separata* developmental stages, but the relative *MsTPS* expression level was lowest in the first instar larvae. Additionally, *MsTPS* was expressed at significantly higher levels in the prepupal, pupal, and adult stages than in the larval stages. The peak *MsTPS* expression level, which was detected in the pupal stage, was 107.76-fold and 1.19-fold higher than the corresponding expression levels in the first instar larval stage and the adult stage, respectively (Figure 1A).

To explore the *MsTPS* expression pattern in diverse *M. separata* tissues, the fourth instar larvae were dissected to obtain the following seven tissues: foregut, midgut, hindgut, fat body, salivary gland, Malpighian tubules, and integument. The results of the qRT-PCR analysis indicated *MsTPS* was expressed in all examined tissues. The relative *MsTPS* expression level was lowest in the hindgut. The *MsTPS* expression levels were significantly higher in the Malpighian tubules and integument than in the foregut, midgut, hindgut, and salivary gland. The *MsTPS* expression level in the integument was higher than that in the Malpighian tubules, but this difference was not significant. The highest *MsTPS* expression level, which was detected in the fat body, was 3.51-fold and 134.87-fold higher than the corresponding expression levels in the integument and the hindgut, respectively (Figure 1B).

### 3.4 Post-RNAi *MsTPS* silencing efficiency

**FIGURE 2:**
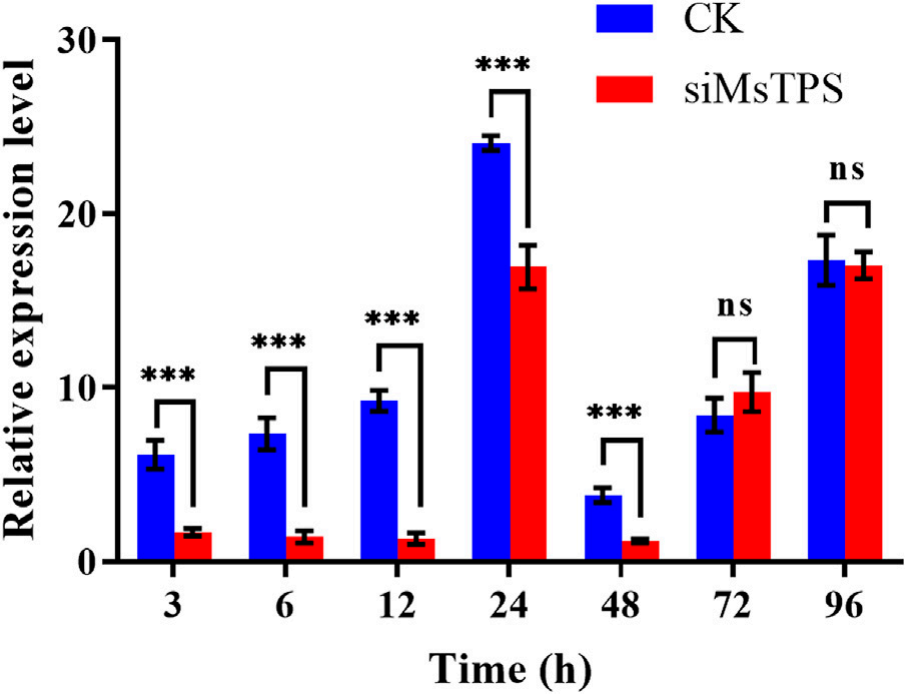
Expression of *MsTPS* at different post-RNAi time-points. The relative *MsTPS* expression levels were determined by qRT-PCR and the 2^−ΔΔCT^ data analysis method. The expression data are provided as the mean ± SE. Data were normalized against the expression data for two reference genes (encoding *β*-actin and glyceraldehyde-3-phosphate dehydrogenase). Statistical analyses were performed using the GraphPad Prism software. Statistically significant differences by *t*-test at same treatment time shown as asterisks (*p < 0.05, **p < 0.01, ***p < 0.001, ns > 0.05).

To assess how efficiently *MsTPS* was silenced by RNAi, the fourth instar larvae were injected with equal amounts of siRNA for *MsTPS* and the negative control. The *MsTPS* expression level was analyzed at 3, 6, 12, 24, 48, 72, and 96 h after the injection. At the 3, 6, 12, 24, and 48 h time-points, the *MsTPS* expression levels were extremely significantly lower in the treated group than in the control group, with inhibition rates of 72.48%, 86.46%, 85.80%, 29.58%, and 69.35%, respectively. Although the *MsTPS* expression levels increased at 72 and 96 h, there was no significant difference in the expression levels of the treated and control groups (Figure 2). Therefore, *MsTPS* was most efficiently silenced at 6 h post-injection.

### 3.5 Changes in the TPS activity and trehalose content after the silencing of *MsTPS*

**FIGURE 3:**
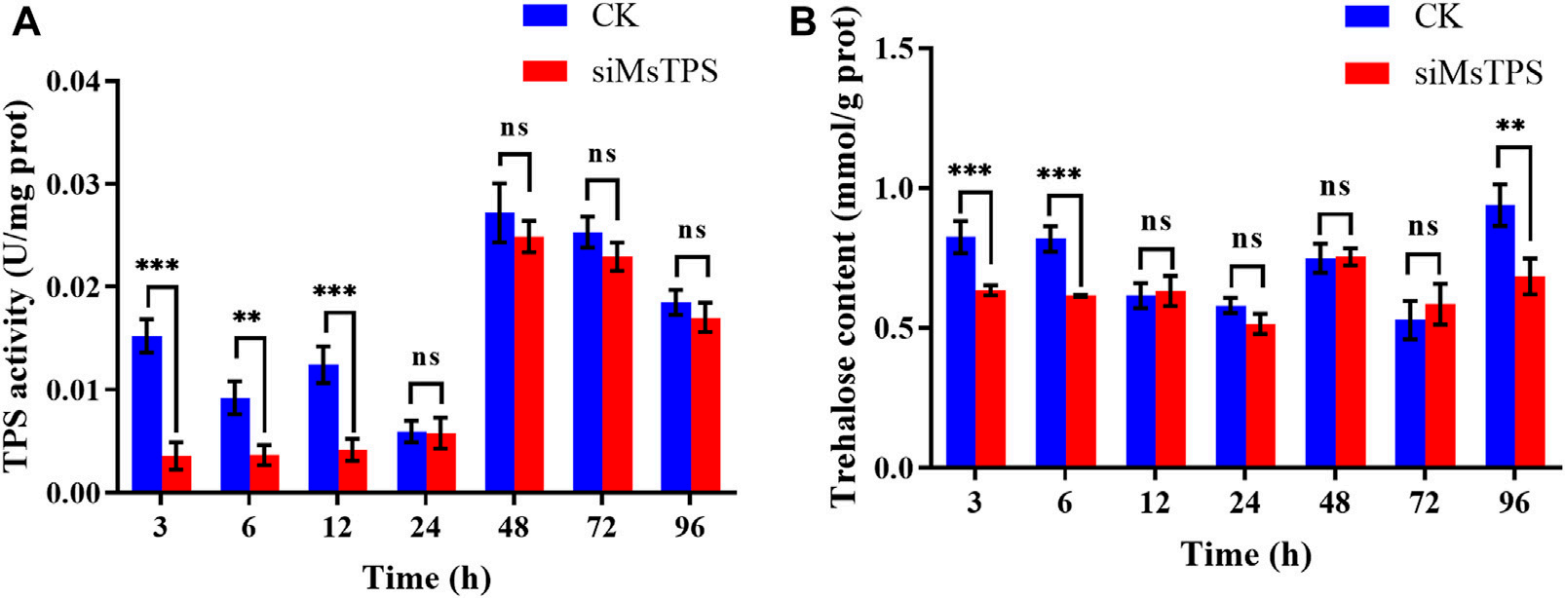
*M. separata* TPS activity **(A)** and trehalose content **(B)** at different time-points after the *MsTPS* RNAi treatment. Data are presented as the mean ± SE. Statistical analyses were performed using the GraphPad Prism software. Statistically significant differences by *t*-test at same treatment time shown as asterisks (*p < 0.05, **p < 0.01, ***p < 0.001, ns > 0.05).

To functionally characterize *MsTPS*, the TPS activity and trehalose content in *M. separata* were analyzed at 3, 6, 12, 24, 48, 72, and 96 h post-RNAi treatment. Compared with the control, the TPS activity at 3, 6, and 12 h post-RNAi treatment decreased by 76.52%, 52.86%, and 54.22%, respectively. In contrast, there were no significant differences in TPS activity between the treated and control groups at the 24, 48, 72, and 96 h time-points (Figure 3A). Compared with the control level, the trehalose content decreased extremely significantly when *MsTPS* was silenced for 3, 6, and 96 h (decreased by 23.17%, 24.88%, and 27.15%, respectively). There were no significant differences in the trehalose content between the treated and control groups at the 12, 24, 48, and 72 h time-points (Figure 3B).

### 3.6 Analysis of *chitin synthase* gene expression and chitin content after the silencing of *MsTPS*

**FIGURE 4:**
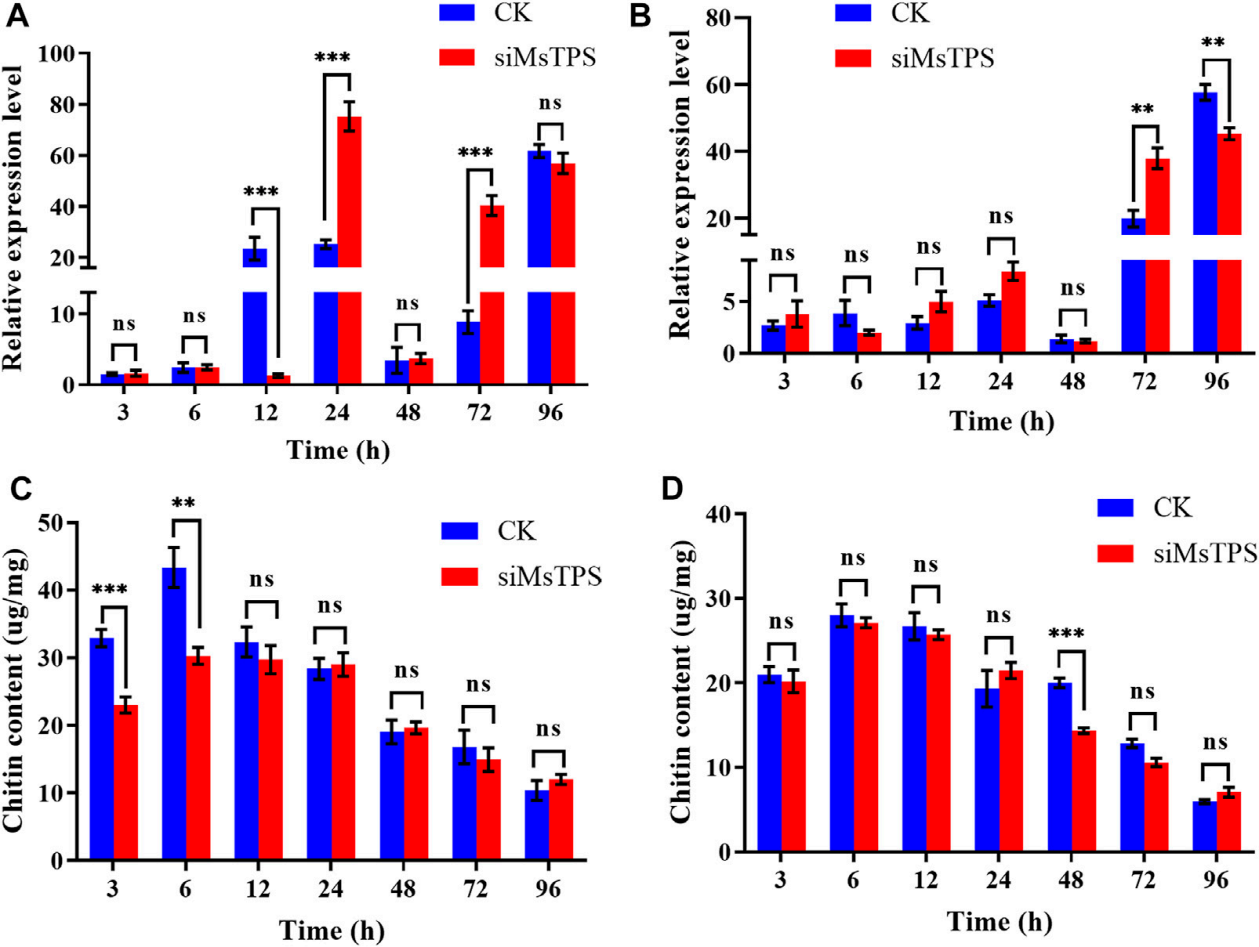
Effect of the silencing of *MsTPS* on *chitin synthase* gene expression and the chitin content. **(A)** Expression of *MsCHSA* at different time-points after the *MsTPS* RNAi treatment. **(B)** Expression of *MsCHSB* at different time-points after the *MsTPS* RNAi treatment. **(C)** Chitin content in the *M. separata* integument at different time-points after the *MsTPS* RNAi treatment. **(D)** Chitin content in the *M. separata* midgut at different time-points after the *MsTPS* RNAi treatment. Relative *MsTPS* expression levels were determined by qRT-PCR and the 2^−ΔΔCT^ data analysis method. Expression data are provided as the mean ± SE. Data were normalized against the expression data for two reference genes (encoding *β*-actin and glyceraldehyde-3-phosphate dehydrogenase). Statistical analyses were performed using the GraphPad Prism software. Statistically significant differences by *t*-test at same treatment time shown as asterisks (*p < 0.05, **p < 0.01, ***p < 0.001, ns > 0.05).

To investigate whether the silencing of *MsTPS* affected the expression of *MsCHS*, larvae were collected at 3, 6, 12, 24, 48, 72, and 96 h after the RNAi treatment for an examination of *MsCHSA* and *MsCHSB* expression. At the 12 h time-point, *MsCHSA* was expressed at a significantly lower level in the treated group than in the control group, and the silencing of *MsTPS* significantly increased the *MsCHSA* expression at the 24 and 72 h time-points (Figure 4A). In addition, the silencing of *MsTPS* significantly increased the *MsCHSB* expression at 72 h time-point and significantly decreased at 96 h time-point (Figure 4B). The results implying that inhibited *MsTPS* expression adversely affected *MsCHS* expression.

To elucidate the effect of *MsTPS* on the *M. separata* chitin content, the larvae were dissected at 3, 6, 12, 24, 48, 72, and 96 h after the RNAi treatment to obtain the midgut and integument, which were subsequently examined regarding their chitin contents. The results showed that the silencing of *MsTPS* extremely significantly decreased the integument chitin content by 29.99% and 30.12% at the 3 and 6 h time-points, respectively (Figure 4C). Additionly, the silencing of *MsTPS* significantly decreased the midgut chitin content by 28.31% at 48 h time-point (Figure 4D).

### 3.7 Effects of the silencing of *MsTPS* on the ability of *M. separata* to utilize food

To investigate the effect of the silencing of *MsTPS* on the ability of *M. separata* to utilize food, the larvae at 3, 6, 12, 24, 48, 72, and 96 h after the RNAi treatment were examined regarding specific nutritional indices. The silencing of *MsTPS* resulted in a significant decrease in the *M. separata* dry weight at 96 h post-RNAi treatment. Moreover, the larval feed intake significantly decreased at the 6, 12, 24, 48, and 96 h post-RNAi treatment time-points. The silencing of *MsTPS* significantly decreased the relative growth rate and the relative consumption rate at 6 and 96 h. Furthermore, the RNAi treatment also significantly decreased the efficiency of the conversion of ingested food at 6 h, the approximate digestibility at 24 h, and the efficiency of the conversion of digested food at 6, 48, and 96 h Table 1. Accordingly, the silencing of *MsTPS* adversely affected the ability of *M. separata* to utilize food.

**TABLE 1:** Nutritional indices for *M. separata* at different time-points after the silencing of *MsTPS* by RNA interference.

**Different treatments**	**Increase in dry weight (g)**	**Larval feed intake (g)**	**Relative growth rate (RGR) [g∙(g∙h)^−1^]**	**Relative consumption rate (RCR) [g∙(g∙h)^−1^]**	**Efficiency of conversion of digested food (ECD) (%)**	**Efficiency of conversion of ingested food (ECI) (%)**	**Approximate digestibility (AD) (%)**
CK 3 h	0.0011 ± 0.0001	0.0041 ± 0.0004	0.0272 ± 0.0028	0.0665 ± 0.0042	47.8742 ± 3.7111	45.2026 ± 3.4430	34.9422 ± 1.5141
si*MsTPS* 3 h	0.0012 ± 0.0001	0.0034 ± 0.0002	0.0310 ± 0.0025	0.0936 ± 0.0112	36.6965 ± 1.0191	37.1864 ± 5.1471	17.2634 ± 2.6476
CK 6 h	0.0032 ± 0.0004	0.0075 ± 0.0002	0.0533 ± 0.0017	0.1313 ± 0.0030	62.0886 ± 1.8594	40.5954 ± 0.6848	44.2561 ± 3.0418
si*MsTPS* 6 h	0.0019 ± 0.0003	0.0047 ± 0.0003**	0.0358 ± 0.0007***	0.0845 ± 0.0012***	35.3609 ± 2.1302***	31.6680 ± 0.7596***	49.7342 ± 3.4406
CK 12 h	0.0039 ± 0.0003	0.0175 ± 0.0001	0.0305 ± 0.0034	0.1295 ± 0.0052	60.6653 ± 4.1909	23.3671 ± 2.9765	34.2510 ± 3.7892
si*MsTPS* 12 h	0.0029 ± 0.0001	0.0126 ± 0.0003***	0.0285 ± 0.0006	0.1238 ± 0.0052	65.4516 ± 3.9655	23.1928 ± 1.3121	34.6162 ± 4.8562
CK 24 h	0.0056 ± 0.0006	0.0232 ± 0.0007	0.0224 ± 0.0027	0.0862 ± 0.0058	51.5945 ± 6.8708	29.3519 ± 2.6503	33.1562 ± 1.4946
si*MsTPS* 24 h	0.0043 ± 0.0008	0.0187 ± 0.0006*	0.0184 ± 0.0038	0.0922 ± 0.0147	37.6385 ± 3.7035	21.7770 ± 3.2105	16.9277 ± 0.0248***
CK 48 h	0.0090 ± 0.0005	0.0469 ± 0.0016	0.0180 ± 0.0015	0.0874 ± 0.0074	68.0102 ± 2.2610	20.7333 ± 1.1907	32.2014 ± 1.7239
si*MsTPS* 48 h	0.0096 ± 0.0008	0.0383 ± 0.0010*	0.0173 ± 0.0001	0.0723 ± 0.0037	52.9722 ± 1.6787**	24.5142 ± 1.4579	31.6346 ± 2.0366
CK 72 h	0.0139 ± 0.0004	0.0661 ± 0.0010	0.0179 ± 0.0010	0.0907 ± 0.0040	63.5514 ± 4.5886	20.1489 ± 0.4596	28.6223 ± 0.6498
si*MsTPS* 72 h	0.0148 ± 0.0009	0.0612 ± 0.0011	0.0187 ± 0.0004	0.0868 ± 0.0016	47.0318 ± 5.9473	22.7698 ± 1.2078	31.8150 ± 2.3600
CK 96 h	0.0387 ± 0.0002	0.1041 ± 0.0021	0.0365 ± 0.0005	0.1021 ± 0.0013	75.1708 ± 1.5756	38.7818 ± 0.9244	14.1180 ± 0.7698
si*MsTPS* 96 h	0.0292 ± 0.0011**	0.0788 ± 0.0036**	0.0272 ± 0.0009**	0.0669 ± 0.0053**	49.2176 ± 1.7186***	42.4051 ± 4.0393	11.4601 ± 1.2654

Data are presented as the mean ± SE. Statistical analyses were performed using the GraphPad Prism software. Statistically significant differences by *t*-test at same treatment time shown as asterisks (*p < 0.05, **p < 0.01, ***p < 0.001).

### 3.8 Effects of the silencing of *MsTPS* on *M. separata* growth and development

**FIGURE 5:**
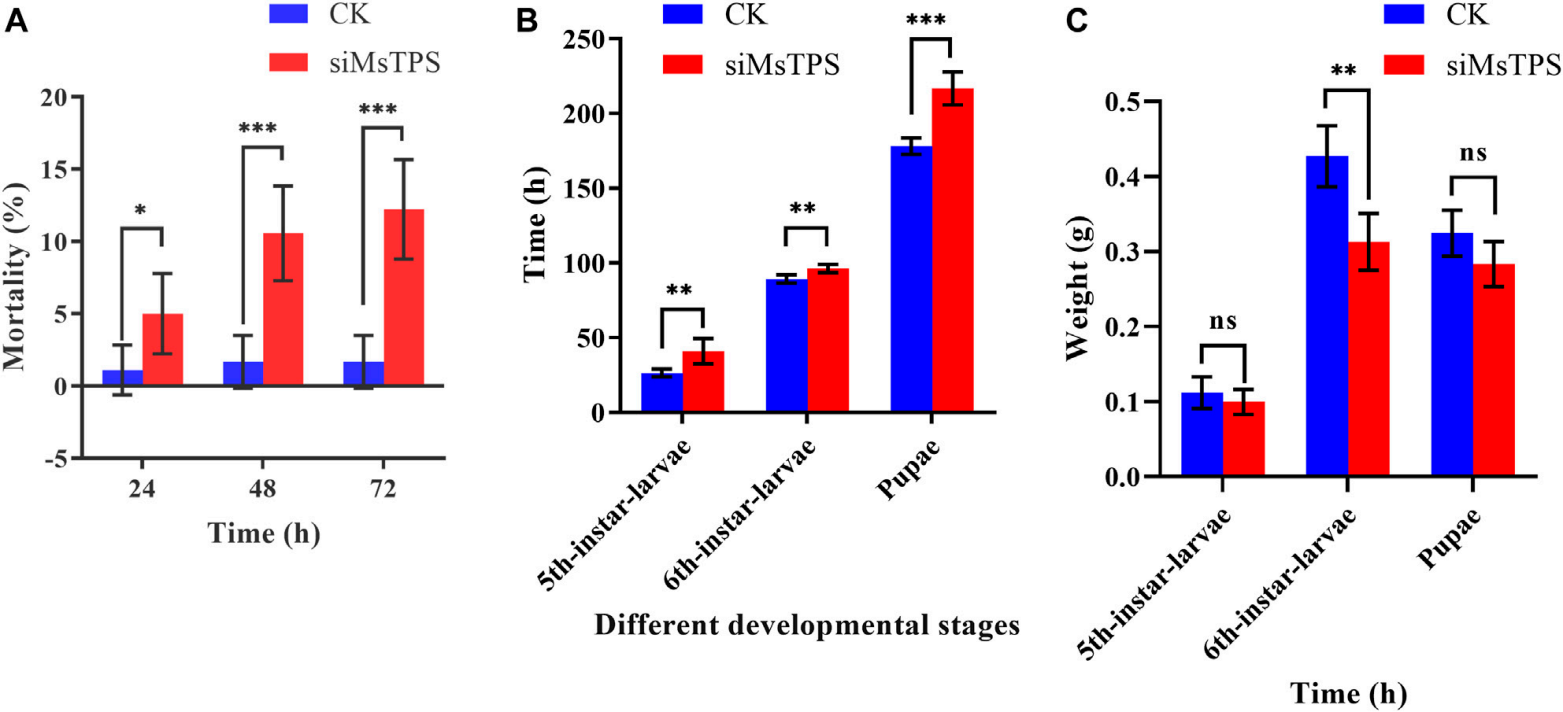
Effect of the silencing of *MsTPS* on *M. separata* mortality **(A)**, developmental time **(B)**, and weight **(C)**. Data are presented as the mean ± SE. Statistically significant differences by *t*-test at same treatment time shown as asterisks (*p < 0.05, **p < 0.01, ***p < 0.001, ns > 0.05).

**FIGURE 6:**
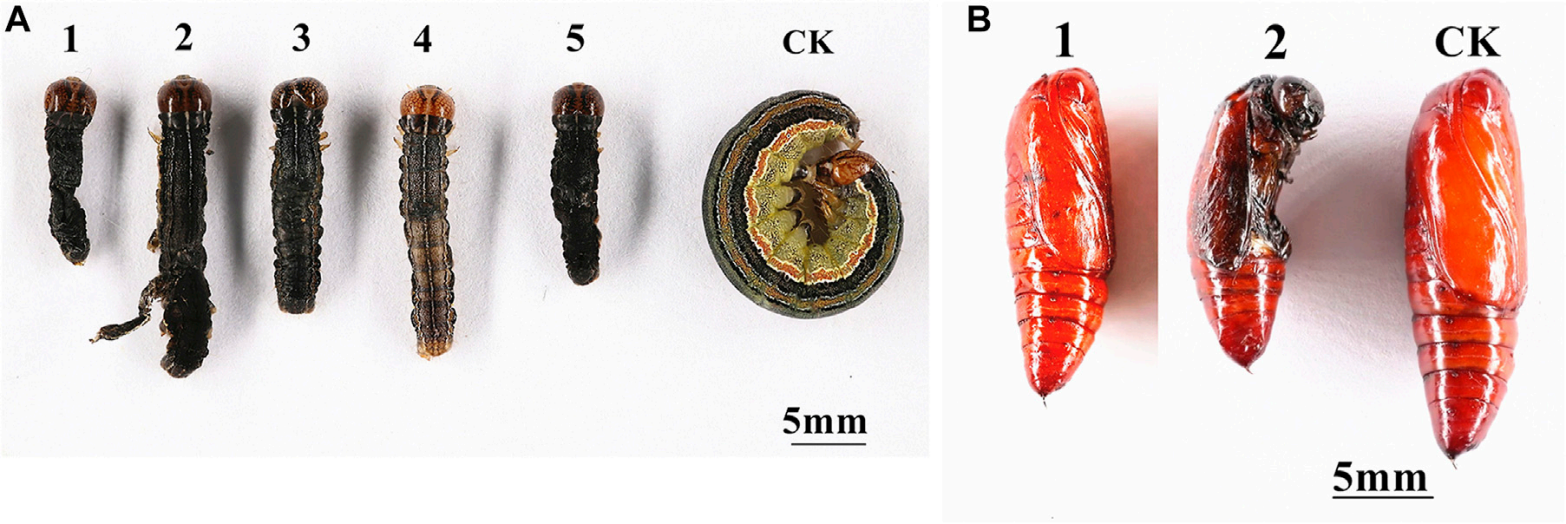
Effects of the silencing of *MsTPS* on *M. separata* molting **(A)** and pupation **(B)**. **(A)** 1, 3, and 5: larvae were unable to molt normally before dying; 2: larvae retained the cuticle from the fourth instar larvae; 4: larvae were too small after molting. **(B)** 1: larvae failed to pupate; 2: pupae were too small.

To determine the effect of the silencing of *MsTPS* on growth and development, the *M. separata* growth and molting processes following the RNAi treatment were analyzed. The silencing of *MsTPS* resulted in a significant increase in the *M. separata* mortality rate at 24, 48, and 72 h (Figure 5A). The RNAi treatment also significantly prolonged the time required for *M. separata* larvae to develop into 5th-instar larvae, 6th-instar larvae, and pupae (Figure 5B). Furthermore, the RNAi treatment also resulted in a significant decrease in the weight of the sixth instar larvae (Figure 5C). Additionally, compared with the control samples, three distinct phenotypic differences were evident in the *M. separata* in which *MsTPS* was silenced (Figure 6A). First, larvae were unable to molt normally before dying. Second, partially deformed fifth instar larvae retained the cuticle from the fourth instar larvae. Third, some larvae were too small after molting. Moreover, *M. separata* pupation abnormalities were detected (Figure 6B). More specifically, some larvae failed to pupate and some pupae were too small. The rates of abnormal molting and pupation were 7.33% and 12.67%, respectively. The phenotypes of the control samples were relatively consistent.

## 4 Discussion

As a stress protectant, reserve carbohydrate, transport sugar, stress-related metabolite, and energy source, trehalose affects insect growth and development, molting, flying, overwintering, and other life cycle-related activities (Delorge et al., 2015). making it the most important carbohydrate in insect hemolymphs. In insects, trehalose is primarily synthesized in the TPS/TPP pathway. In this study, the *M. separata TPP* sequence was not detected in the screened transcriptome database. Earlier research revealed that TPS alone can mediate trehalose synthesis (Tang et al., 2018).

The *H. armigera HaTPS* gene is reportedly expressed during all developmental stages, with peak expression in the late sixth larval instar stage (Xu et al., 2009). In the current study, *MsTPS* was expressed in all examined *M. separata* developmental stages, with the lowest and highest expression levels detected in the first instar larvae and pupae, respectively.

The *MsTPS* expression pattern detected in this study was basically consistent with the published expression data for other insects. For example, in *L. migratoria*, *LmTPS* is reportedly mainly expressed in the fat body, but it is also expressed at low levels in the muscle tissue, hemolymph, and intestinal tract (Cui and Xia, 2009). In insects, trehalose is produced in fat body, which is also where *TPS* is mainly expressed (Chung, 2008; Tang et al., 2010). Trehalose, which is considered to be a source of stored energy and carbon, provides energy for insect growth and development, molting, and flying (Chung, 2008). The differential expression of *TPS* can affect the trehalose content in insects (Chen et al., 2010). Because TPS is a key enzyme in the trehalose synthesis pathway, it substantially affects trehalose production. In arthropods, *TPS* expression directly affects the trehalose content (Shi and Chung, 2014). In the current study, *MsTPS* expression was inhibited *via* RNAi, which resulted in changes to TPS activity and the trehalose content in *M. separata*.

Insect molting requires chitin synthesis and degradation pathways (Zhu et al., 2016). Trehalose is a substrate for insect chitin synthesis pathways. Studies have demonstrated that an imbalance in the trehalose synthesis and degradation in insects is associated with changes in the expression levels of related genes in the chitin synthesis pathway, with significant decreases in the chitin contents resulting in molting abnormalities and high mortality rates (Tang et al., 2016; Zhao et al., 2016). Moreover, suppressed *TPS* expression in insects leads to abnormal molting and death (Tang et al., 2016; Yang et al., 2017; Chen et al., 2018). In the current study, the silencing of *MsTPS* significantly affected *MsCHSA* and *MsCHSB* expression and significantly decreased the integument and midgut chitin content. Additionly, the RNAi treatment significantly increased the *M. separata* mortality rate and the time required for larvae and pupae to develop, whereas it significantly decreased the weight of the sixth instar larvae, while also leading to abnormal molting and pupation. These findings are in accordance with the results of earlier analyses of other insects. In previous investigations involving *Nilaparvata lugens*, inhibited *NlTPS1* expression resulted in malformation and mortality rates of 20% and 30%, respectively (Chen et al., 2010; Yang et al., 2017). In another study, decreases in the *TPS* expression level in *S. exigua* led to mortality rates of 50.94% and 66.76% at 48 and 204 h, respectively (Tang et al., 2010). Additionally, injecting the third instar larvae of *B. minax* with dsRNA for *BmTPS* reportedly results in abnormal phenotypic changes and a mortality rate of 52% (Xiong et al., 2016). Our results are also in keeping with previous research showing that inhibition of *TPS* gene can lead to malformation and increased mortality rate of insects.

Previous research on *L. decemlineata* revealed that compared with the controls, insects in which *LdTPS* expression is inhibited consume more leaves and are heavier, but they have less chitin (Shi et al., 2016). In the present study, the silencing of *MsTPS* had significant detrimental effects on the ability of *M. separata* to utilize food. These findings were not completely consistent with the reported results for *L. decemlineata*. After the RNAi-based silencing of *MsTPS*, the *M. separata* dry weight and larval feed intake decreased significantly, but the change in the chitin content was consistent with that detected in *L. decemlineata*. An earlier study indicated that the trehalose concentration in insects can modulate insect feeding behaviors (Thompson, 2003). In this study, the silencing of *MsTPS* in *M. separata* resulted in a significant decrease in the trehalose content, thus the decrease of the ability to utilize food which may be related to the decrease in the trehalose content. In this study, it was found the chitin content in the peritrophic membrane significantly decreased in response to the silencing of *MsTPS*. The significant decrease in the ability of *M. separata* to utilize food might be associated with alterations to the peritrophic membrane structure and decreases in the chitin content, but this will need to be experimentally verified.

## Data Availability

The datasets presented in this study can be found in online repositories. The names of the repository/repositories and accession number(s) can be found below: The MsTPS cloned in this study has been recorded into NCBI repository. GenBank accession number: MN832898. https://www.ncbi.nlm.nih.gov/nuccore/MN832898.1/; Transcriptome Sequencing clean reads were submitted to NCBI SRA database: PRJNA919163.

## References

[B1] Arakane Y. Muthukrishnan S. Kramer K. J. Specht C. A. Tomoyasu Y. Lorenzen M. D. (2005). The *Tribolium chitin synthase* genes *TcCHS1* and *TcCHS2* are specialized for synthesis of epidermal cuticle and midgut peritrophic matrix. Insect Mol. Biol. 14 (5), 453–463. 10.1111/j.1365-2583.2005.00576.x 16164601

[B2] Bradford M. M. (1976). A rapid and sensitive method for the quantitation of microgram quantities of protein utilizing the principle of protein-dye binding. Anal. Biochem. 72 (1-2), 248–254. 10.1006/abio.1976.9999 942051

[B3] Chen J. X. Lu Z. H. Wang C. Y. Chen J. Lin T. (2020). RNA interference of a *trehalose-6-phosphate synthase* gene reveals its roles in the biosynthesis of chitin and lipids in *Heortia vitessoides* (Lepidoptera: Crambidae). Insect Sci. 27 (2), 212–223. 10.1111/1744-7917.12650 30397994 PMC7379938

[B4] Chen J. Zhang D. Yao Q. Zhang J. Dong X. Tian H. (2010). Feeding-based RNA interference of a *trehalose phosphate synthase* gene in the Brown planthopper. Nil. Lugens. Insect Mol. Biol. 19 (6), 777–786. 10.1111/j.1365-2583.2010.01038.x 20726907

[B5] Chen Q. Ma E. Behar K. L. Xu T. Handan G. G. (2002). Role of trehalose phosphate synthase in anoxia tolerance and development in *Drosophila melanogaster* . J. Biol. Chem. 277 (5), 3274–3279. 10.1074/jbc.M109479200 11719513

[B6] Chen Q. W. Jin S. Zhang L. Shen Q. D. Wei P. Wei Z. M. (2018). Regulatory functions of trehalose-6-phosphate synthase in the chitin biosynthesis pathway in *Tribolium castaneum* (Coleoptera: Tenebrionidae) revealed by RNA interference. B. Entomol. Res. 108 (3), 388–399. 10.1017/S000748531700089X 28920565

[B7] Chung J. S. (2008). A *trehalose 6-phosphate synthase* gene of the hemocytes of the blue crab, *Callinectes sapidus*: Cloning, the expression, its enzyme activity and relationship to hemolymph trehalose levels. Saline Syst. 4 (1), 18. 10.1186/1746-1448-4-18 19077285 PMC2615023

[B8] Cui S. Y. Xia Y. X. (2009). Isolation and characterization of the *trehalose-6-phosphate synthase* gene from *Locusta migratoria manilensis* . Insect Sci. 16 (4), 287–295. 10.1111/j.1744-7917.2009.01268.x

[B9] Delorge I. Figueroa C. M. Feil R. Lunn J. E. Van D. P. (2015). Trehalose-6-phosphate synthase 1 is not the only active TPS in *Arabidopsis thaliana* . *Arabidopsis thaliana*. Biochem. J. 466 (2), 283–290. 10.1042/BJ20141322 25495218

[B10] Ge L. Q. Zhao K. F. Huang L. J. Wu J. C. (2011). The effects of triazophos on the trehalose content, trehalase activity and their gene expression in the Brown planthopper *Nilaparvata lugens* (Hemiptera: Delphacidae). Pestic. Biochem. Physiol. 100 (2), 172–181. 10.1016/j.pestbp.2011.03.007 21760647 PMC3102831

[B11] Giaever H. M. Styrvold O. B. Kaasen I. Strom A. R. (1988). Biochemical and genetic characterization of osmoregulatory trehalose synthesis in *Escherichia coli* . J. Bacteriol. 170 (6), 2841–2849. 10.1128/jb.170.6.2841-2849.1988 3131312 PMC211211

[B12] Guo X. Wang Y. Sinakevitch I. Lei H. Smith B. H. (2018). Comparison of RNAi knockdown effect of tyramine receptor 1 induced by dsRNA and siRNA in brains of the honey bee, *Apis mellifera* . J. Insect Physiol. 111, 47–52. 10.1016/j.jinsphys.2018.10.005 30393170

[B13] Hottiger T. Boller T. Wiemken A. (1987). Rapid changes of heat and desiccation tolerance correlated with changes of trehalose content in *Saccharomyces cerevisiae* cells subjected to temperature shifts. FEBS Lett. 220 (1), 113–115. 10.1016/0014-5793(87)80886-4 3301407

[B14] Jo V. Katleen D. P. Filip P. Bruce P. Nadine V. R. Anne D. P. (2002). Accurate normalization of real-time quantitative RT-PCR data by geometric averaging of multiple internal control genes. Genome Biol. 3 (7), RESEARCH0034. 10.1186/gb-2002-3-7-research0034 12184808 PMC126239

[B15] Kumar S. Stecher G. Tamura K. (2016). MEGA7: Molecular evolutionary genetics analysis version 7.0 for bigger datasets. Mol. Biol. Evol. 33 (7), 1870–1874. 10.1093/molbev/msw054 27004904 PMC8210823

[B16] Mole S. Zera A. J. (1993). Differential allocation of resources underlies the dispersal-reproduction trade-off in the wing-dimorphic cricket, *Gryllus rubens* . Oecologia 93 (1), 121–127. 10.1007/BF00321201 28313784

[B17] Reissig J. L. Strominger J. L. Leloir L. F. (1955). A modified colorimetric method for the estimation of N-acetylamino sugars. J. Biol. Chem. 217 (2), 959–966. 10.1016/s0021-9258(18)65959-9 13271455

[B18] Santos R. Alves-Bezerra M. Rosas-Oliveira R. Majerowicz D. Meyer-Fernandes J. R. Gondim K. C. (2012). Gene identification and enzymatic properties of a membrane-bound trehalase from the ovary of *Rhodnius prolixus* . Arch. Insect Biochem. Physiol. 81 (4), 199–213. 10.1002/arch.21043 22851503

[B19] Shi J. F. Xu Q. Y. Sun Q. K. Meng Q. W. Mu L. L. Guo W. C. (2016). Physiological roles of trehalose in Leptinotarsa larvae revealed by RNA interference of *trehalose-6-phosphate synthase* and *trehalase* genes. Insect biochem. Mol. Biol. 77, 52–68. 10.1016/j.ibmb.2016.07.012 27524277

[B20] Shi Q. Chung J. S. (2014). Trehalose metabolism in the blue crab *Callinectes sapidus*: Isolation of multiple structural cDNA isoforms of *trehalose-6-phosphate synthase* and their expression in muscles. Gene 536 (1), 105–113. 10.1016/j.gene.2013.11.070 24334121

[B21] Shukla E. Thorat L. J. Nath B. B. Gaikwad S. M. (2015). Insect trehalase: Physiological significance and potential applications. Glycobiology 25 (4), 357–367. 10.1093/glycob/cwu125 25429048

[B22] Song J. C. Lu Z. J. Yi L. Yu H. Z. (2021). Functional Characterization of a *trehalose-6-phosphate synthase* in *Diaphorina citri* revealed by RNA interference and transcriptome sequencing. Insects 12 (12), 1074. 10.3390/insects12121074 34940162 PMC8709273

[B23] Steele J. E. (1988). Occurrence of a hyperglycæmic factor in the corpus cardiacum of an insect. Nature 192 (4803), 680–681. 10.1038/192680a0

[B24] Strom A. R. Kaasen I. (1993). Trehalose metabolism in *Escherichia coli*: Stress protection and stress regulation of gene expression. Mol. Microbiol. 8 (2), 205–210. 10.1111/j.1365-2958.1993.tb01564.x 8391102

[B25] Tang B. Chen J. Yao Q. Pan Z. Q. Xu W. H. Wang S. G. (2010). Characterization of a *trehalose-6-phosphate synthase* gene from *Spodoptera exigua* and its function identification through RNA interference. J. Insect Physiol. 56 (7), 813–821. 10.1016/j.jinsphys.2010.02.009 20193689

[B26] Tang B. Qin Z. Shi Z. K. Wang S. Guo X. J. Wang S. G. (2014). Trehalase in *Harmonia axyridis* (Coleoptera: Coccinellidae): Effects on beetle locomotory activity and the correlation with trehalose metabolism under starvation conditions. Appl. Entomol. Zool. 49 (2), 255–264. 10.1007/s13355-014-0244-4

[B27] Tang B. Wang S. Wang S. G. Wang H. J. Zhang J. Y. Cui S. Y. (2018). Invertebrate *trehalose-6-phosphate synthase* gene: Genetic architecture, biochemistry, physiological function, and potential applications. Front. Physiol. 9, 30. 10.3389/fphys.2018.00030 29445344 PMC5797772

[B28] Tang B. Wei P. Zhao L. N. Shi Z. K. Shen Q. D. Yang M. M. (2016). Knockdown of five *trehalase* genes using RNA interference regulates the gene expression of the chitin biosynthesis pathway in *Tribolium castaneum* . Bmc Biotechnol. 16 (1), 67. 10.1186/s12896-016-0297-2 27596613 PMC5011928

[B29] Thompson S. N. (2003). Trehalose-the insect ‘blood’ sugar. Adv. Insect Physiol. 31, 205–285. 10.1016/S0065-2806(03)31004-5

[B30] Waldbauer G. P. (1968). The consumption and utilization of food by insects. Adv. Insect Physiol. 5, 229–288. 10.1016/s0065-2806(08)60230-1

[B31] Xiong K. C. Wang J. Li J. H. Deng Y. Q. Pu P. Fan H. (2016). RNA interference of a *trehalose-6-phosphate synthase* gene reveals its roles during larval-pupal metamorphosis in *Bactrocera minax* (Diptera: Tephritidae). J. Insect Physiol. 91-92, 84–92. 10.1016/j.jinsphys.2016.07.003 27405007

[B32] Xu J. Bao B. Zhang Z. F. Yi Y. Z. Xu W. H. (2009). Identification of a novel gene encoding the trehalose phosphate synthase in the cotton bollworm, *Helicoverpa armigera* . Glycobiology 19 (3), 250–257. 10.1093/glycob/cwn127 19004876

[B33] Yang M. M. Zhao L. N. Shen Q. D. Xie G. Q. Wang S. G. Tang B. (2017). Knockdown of two *trehalose-6-phosphate synthases* severely affects chitin metabolism gene expression in the Brown planthopper *Nilaparvata lugens* . Pest Manag. Sci. 73 (1), 206–216. 10.1002/ps.4287 27060284

[B34] Zhang Z. Zhang Y. H. Wang J. Liu J. Tang Q. B. Li X. R. (2018). Analysis on the migration of first-generation *Mythimna separata* (Walker) in China in 2013. J. Integr. Agric. 17 (7), 1527–1537. 10.1016/S2095-3119(17)61885-9

[B35] Zhao L. N. Yang M. M. Shen Q. D. Liu X. J. Shi Z. K. Wang S. G. (2016). Functional characterization of three *trehalase* genes regulating the chitin metabolism pathway in rice Brown planthopper using RNA interference. Sci. Rep. 6, 27841. 10.1038/srep27841 27328657 PMC4916506

[B36] Zhao L. Q. Liao H. Y. Zeng Y. Wu H. J. Zhu D. H. (2017). Food digestion capability and digestive enzyme activity in female adults of the wing-dimorphic cricket *Velarifictorus ornatus* . Entomol. Exp. Appl. 163 (1), 35–42. 10.1111/eea.12563

[B37] Zhu K. Y. Merzendorfer H. Zhang W. Q. Zhang J. Z. Muthukrishnan S. (2016). Biosynthesis, turnover, and functions of chitin in insects. Annu. Rev. Entomol. 61, 177–196. 10.1146/annurev-ento-010715-023933 26982439

