# Supplementary material for: Trehalose-6-phosphate synthase regulates chitin synthesis in Mythimna separata
Source: Front Physiol. 2023 Feb 13;14:1109661. doi: 10.3389/fphys.2023.1109661 (PMC9968958; doi:10.3389/fphys.2023.1109661)
Supplement: Supplementary file 1 [file Table1.DOCX]

**Table 1.** Details regarding the primers used in this study

| **Primer Name** | **Primer sequence (5'-3')** | **Usage** |
| --- | --- | --- |
| *MsTPS*-F | GTGATTGGCCGTGAATAGGAA | cDNA cloning |
| *MsTPS*-R | ATCGGCGGAGCGTTTC |  |
| *MsTPS*-q-F | GAACAGGCGAGGAAGCTCAT | qRT-PCR |
| *MsTPS*-q-R | ACTCCAGTCCAATCCGAACG |  |
| *Msβ-actin*-q-F | CCAACGGCATCCACGAGACCA |  |
| *Msβ-actin*-q-R | TCGGCGATACCAGGGTACAT |  |
| *MsGAPDH*-q-F | GCTACAGTCGTTGCCATCAA |  |
| *MsGAPDH*-q-R | GAGGACGGAGATTTTGTTGC |  |
| *MsCHSA*-q-F | GGTGATACAGTTCTCCGCTATG |  |
| *MsCHSA*-q-R | CTAGCAGAGCGTCTTGAGATAAG |  |
| *MsCHSB*-q-F | TCCAGGAGGAACACCATACA |  |
| *MsCHSB*-q-R | GTCAGCCTCCTCTTGAAGTTAG |  |
