# Supplementary figures and images for: Trehalose-6-phosphate synthase regulates chitin synthesis in Mythimna separata
Source: Front Physiol. 2023 Feb 13;14:1109661. doi: 10.3389/fphys.2023.1109661 (PMC9968958; doi:10.3389/fphys.2023.1109661)

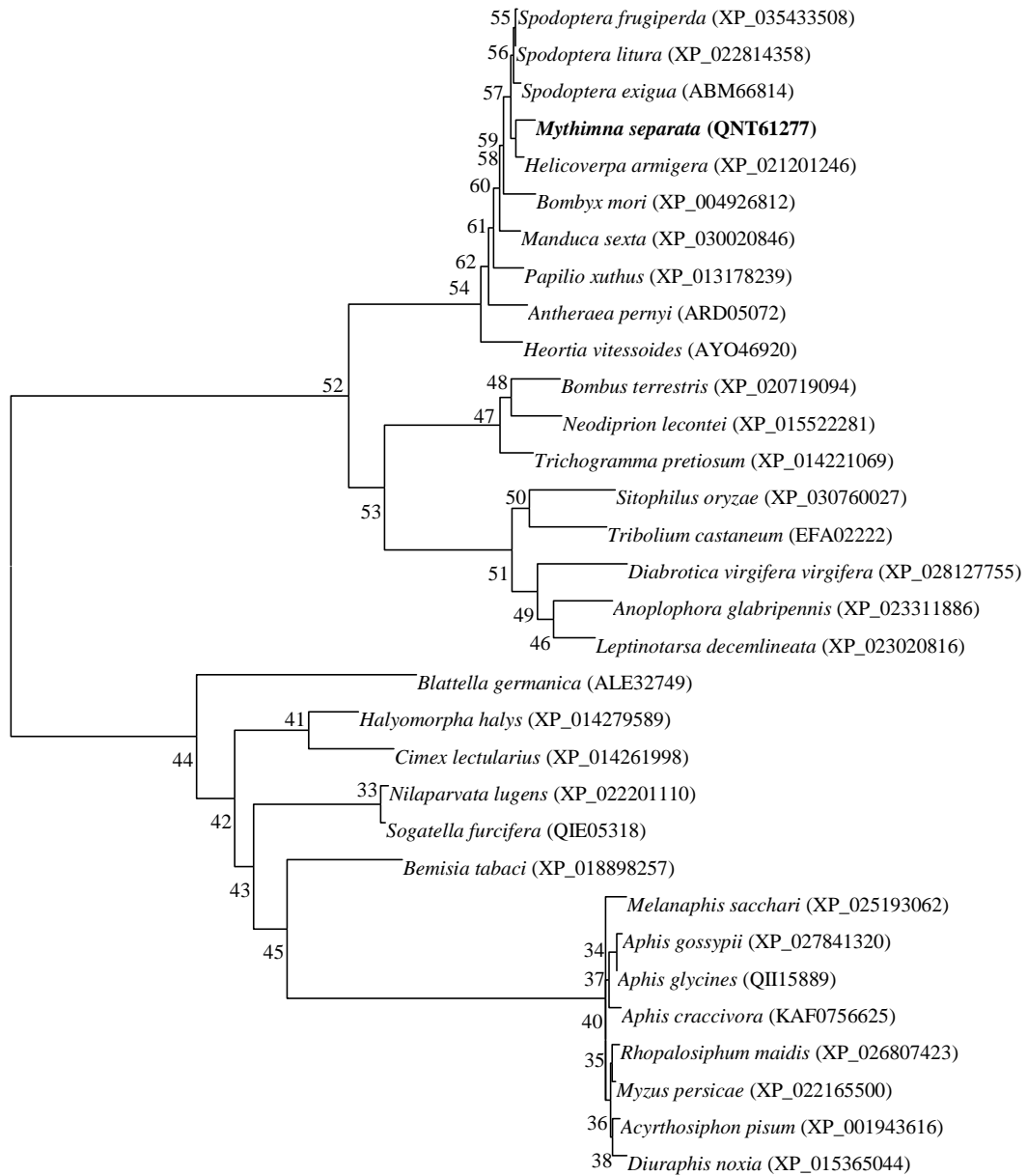

0.10

Supplement: Supplementary file 2 [file DataSheet1.PDF]
